# The Reaction of Degeneration

**Published:** 1909-01-02

**Authors:** 


### Medicine.

### THE REACTION OF DEGENERATION.

Before any account of the reaction of degeneration can be clearly understood it is essential to have a clear conception first of the effect of electricity on normal muscles and nerves, and secondly of the histological changes that occur in nerves and in muscles when the former undergo degeneration.

### Effect of Electricity upon Normal Muscles and Nerves.

For clinical purposes two forms of electric current are employed in diagnosis, the continuous or galvanic, and the interrupted or faradic. Both these forms, when applied in sufficient strength either to healthy motor nerves or to healthy muscles, evoke contractions in the latter; but whereas with the faradic current the healthy muscle is thrown into continuous contraction, with the galvanic current the muscle remains quite placid and still, except when the strength of the current passing through it is either suddenly increased or suddenly diminished. A sufficient amount of a constant current may be suddenly run into a muscle or nerve by closing a key in the circuit, and the result will be a single contraction of the muscle, the so-called "closure contraction." The muscle thereafter remains relaxed and motionless so long as the strength of current running through it remains the same, but when that current is suddenly interrupted by opening the key in the circuit there is again a single muscular response—the so-called "opening contraction." It is not absolutely necessary to break the current altogether to get a contraction, but the point is that the mere fact of a constant current flowing through a muscle or nerve does not constitute a stimulus; the current must be suddenly increased or suddenly decreased for stimulation to occur. The simplest conditions for descriptive purposes are when the change is from zero to full strength when the current is closed, and from full strength to zero when the current is opened.

Upon stimulation of either muscle or nerve by a faradic current on the other hand, there is no quiescent interval; a continuous tonic contraction results if muscle and nerve are healthy and if the faradic current is not too weak. The reason for this is that a faradic current is in reality a constant current that is being very rapidly made and broken, with the result that there are very rapidly recurring closure and opening contractions which fuse into one tetanic spasm.

When a constant current is employed, it is usual to have one pole or electrode upon the skin over the muscle or nerve to be stimulated, and the other over the skin of some other part of the body. It is clearly possible for the current under these circumstances either to pass from the battery to the electrode over the muscle, thence into the muscle, and out through the other electrode, and so back to the battery; or to pass from the battery to the electrode which is not over the muscle or nerve, thence into the body, and out through the muscle or nerve to be stimulated, and from the electrode over it back to the battery. In other words, the current may be entering the muscle or nerve from without inwards or from within outwards. The pole or electrode from which the current passes from the battery into the body is termed the *anode*; the pole or electrode from which the current passes from the body back to the battery is termed the *kathode*. In healthy persons there are differences in the minimum strengths of currents required to evoke a contraction according as the current is being either made or broken—*i.e.* closed or opened—on the one hand, or according as the electrode upon the skin over the muscle or nerve to be stimulated is the anode or the kathode upon the other. As a matter of fact, the difference between opening and closure contractions is not made any great use of in clinical medicine, and chief stress is laid upon whether the contraction is obtained more easily with the anode upon the muscle or the kathode. In healthy states, both as regards muscles and as regards nerves, contraction is more readily obtained upon closing the current when the pole upon the muscle is the kathode than when it is the anode. For the sake of brevity this is usually expressed by the formula K.C.C. > A.C.C., which may be briefly expressed in words by saying that the kathodal closure contraction is more easily obtained than is the anodal closure contraction. It is also important to know that the normal muscular response to galvanic excitation is short, sharp, and quick.

### The Changes in the Nerves which cause Reaction of Degeneration.

Without going into great detail, a few words are necessary in regard to these. In the first place, for reaction of degeneration to occur there must be a solution of continuity—temporary or otherwise—of the lower motor neuron at some point between or including the anterior cornual cell and the motor end plates. The break in the continuity of the motor nerve may be due to all sorts of causes—division by trauma for example, or inflammation in the nerve trunk, or acute anterior poliomyelitis in the anterior cornu, or pressure by tumours, callus, splints, and so on, or degeneration pure and simple as in progressive muscular atrophy—and the characters of the reaction of degeneration are the same in all. If the lesion is in the upper neuron there will be paralysis, but no wasting and no reaction of denegeration; if the lesion is functional there may be paralysis, but no wasting and no reaction of degeneration; if the lesion is primarily in the muscles themselves as in pseudo-hypertrophic muscular paralysis or one of the other primary muscular dystrophies, there may be paralysis and extreme wasting, but no reaction of degeneration. In other words, testing for and finding that reaction of degeneration is present in a given case is proof positive not only of organic disease but of organic disease in the anterior cornual cells or motor nerve fibres of the lower neuron.

In this connection it is very important to remember four things in particular. First, the lesion may really be one of the lower neuron, and yet there may be no reaction of degeneration, because temporary inflammation is sufficient to inhibit function and so cause paralysis for the time being, though it is insufficient to cause solution of continuity of the axis cylinders of the motor nerve fibres. This is well exemplified by certain cases of facial paralysis of Bell's lower neuron type. Two patients may present themselves with an identical history of facial paralysis for two or three days. The pathology in each case may be inflammation of the periosteum at the stylo-mastoid foramen compressing the facial nerve at its exit from the skull. But, whereas in the one case there is commencing reaction of degeneration, in the other the electrical reactions are normal. It will be found that in the former case the paralysis will persist for twelve weeks or more before it begins to improve, whereas in the other there may be complete cure within a fortnight. The difference is due to the different degree of the periosteal inflammation in the two cases; in the first it has so strangled the nerve that the axis cylinders are broken through, and recovery of function can only occur when fresh axis cylinders have grown down from the central end of the nerve; whilst in the second there is just enough compression of the nerve to inhibit the passage of voluntary impulses, and yet not enough to divide the axis cylinders, so that the latter were able to perform their normal functions again as soon as the periosteal inflammation had more or less subsided.

Secondly, it takes a certain number of days for reaction of degeneration to develop, even when the lower neuron is interrupted; it is not to be expected in a nerve that has been cut through but the moment before.

Thirdly, it is only when all the motor nerve fibres going to a muscle are degenerated that all the points of a typical reaction of degeneration are to be found. In the great majority of cases, however, there are a smaller or a larger number of undegenerated nerve fibres left amongst the degenerated ones in the motor nerve. This is a point upon which far too little stress is laid as a rule; it is clear that the normal fibres will respond in a normal manner, and that the degenerated fibres will respond in the abnormal manner, with the result that a mixed or partial reaction of degeneration will be obtained. Nor will the partial reaction of degeneration in one case be the same as the partial reaction of degeneration in another. If one may take a hypothetical instance in which a motor nerve contained exactly one hundred fibres, it might happen in one patient that ten of these were degenerated and ninety were still normal; in another patient fifty might be normal and fifty degenerated; whilst in another ninety might be degenerated and only ten normal, and so on. The degree of partial reaction of degeneration will be very different in these different cases. It is clear that in cases such as those of progressive muscular atrophy where there is a very slow advance of the degenerative process from one anterior cornual cell to the next there must be an equally slow change from the normal to the typical abnormal electrical reactions passing through every phase of partial reaction of degeneration on the way.

Fourthly, if the lesion of the lower neuron is not recovered from, a time will come when all the muscular fibres attached to degenerated nerve fibres have become converted into inert fibrous tissue. When this is so, no reaction of degeneration is to be obtained at all; either every single fibre in the muscle has become fibrous, in which case no electrical stimulation will evoke any response or reaction at all; or else a few normal fibres will still be left, owing to some of the nerve fibres having escaped degeneration, in which case the electrical responses or reactions of the nerve and muscle will be less strong than they formerly were, but they will otherwise be absolutely normal.

### Complete Reaction of Degeneration.

We are now in a position to give the various points that go to make up a "complete" reaction of degeneration:—

(1) Stimulation of the motor nerve produces no muscular contractions, no matter whether the faradic or the galvanic current is employed.

(2) The paralysed muscle cannot be made to contract by faradic excitation. This is probably due to the fact that with faradism the current is made and broken with such rapidity that the comparatively coarse elements of a muscle are incapable of being stimulated by so fine a stimulus. It seems likely that when a normal muscle is made to contract by a faradic current applied directly over it, the parts that are primarily stimulated are the sensitive nerve endings in the muscle and not the muscle fibres themselves.

(3) When the muscle is directly stimulated by the continuous current, contractions are produced at make and at break as before; but there are three points in which the contractions differ from those evoked in a normal muscle, and these are:—

(*a*) They are more readily excited; that is to say, the muscle fibre whose nerve is degenerating is caused to contract by a smaller galvanic current than the minimum required to cause a contraction in the same muscle in health.

(*b*) The character of the muscular contraction is altered; instead of being short, sharp, and sudden, as in health, it is slow and sluggish, and often very considerably delayed as well.

(*c*) The order of the polar actions is altered, the anodal closure contraction (A.C.C.) tending to become equal to or even greater than the kathodal closure contraction (K.C.C.).

